# Effective refractive error coverage: an eye health indicator to measure progress towards universal health coverage

**DOI:** 10.1111/opo.12662

**Published:** 2019-12-26

**Authors:** Ian McCormick, Islay Mactaggart, Andrew Bastawrous, Matthew J Burton, Jacqueline Ramke

**Affiliations:** ^1^ International Centre for Eye Health Faculty of Infectious and Tropical Diseases London School of Hygiene & Tropical Medicine London UK

## Universal health coverage and eye health

In 2015, all United Nations Member States adopted seventeen Sustainable Development Goals (SDGs), to be achieved by 2030.^1^ One of these – SDG 3 – relates specifically to health, and includes a target (3.8) to “achieve universal health coverage, including financial risk protection, access to quality essential health‐care services and access to safe, effective, quality and affordable essential medicines and vaccines for all.”^1^

Universal health coverage (UHC) means that anyone who needs health care can access quality health services without risk of financial harm.^2^ UHC aspires to include the world's poor and marginalised in health service improvements so that ‘no one is left behind’. Quality‐of‐care is embodied within the concept of UHC and the World Health Organization (WHO) recommends that ‘effective’ coverage indicators are a necessary approach to capture data on quality in monitoring progress in service provision. Effective service coverage describes coverage of sufficient quality to allow for maximum possible health gains.^3^

In the recent *World Report on Vision*, WHO called for the routine measurement of effective coverage of refractive error and effective coverage of cataract surgery as a means to monitor eye health service coverage and quality within UHC.^4^ Cataract and refractive error are the cause of almost three‐quarters of vision impairment (moderate or worse; presenting visual acuity <6/18) globally, affecting an estimated 189 million people in 2015.^5^ Both conditions have efficacious treatment, and the ability to define and measure outcomes with visual acuity after correction or surgery enables an assessment of quality to be made and, therefore, for effective coverage to be calculated.

Effective cataract surgical coverage (eCSC) was defined and its calculation outlined in 2017,^6^ but a similar detailed outline is not yet available for effective refractive error coverage (eREC). For more than a decade, authors have reported ‘refractive error’ or ‘spectacle’ coverage metrics from population‐based surveys^7^, ^8^, ^9^, ^10^, ^11^, ^12^, ^13^, ^14^, ^15^ and, thanks to the visual acuity measurements used in their definitions, these are akin to effective coverage. However, methodological descriptions and definitions have been inconsistent across these surveys, and often relied on assumptions that potentially overestimated the need for correction and subsequent coverage measures. We have reviewed these prior definitions, and here we outline a method to measure and calculate eREC.

## Defining effective refractive error coverage (eREC)

World Health Organization's *World Report on Vision* listed three data points necessary to calculate effective refractive error coverage. In *Table* 1 we provide technical details for these and outline how they equate to measures of met need, under‐met need and unmet need for refractive error correction. Details are outlined below, followed by discussion of measurement and reporting aspects.

We propose that the existing WHO mild distance vision impairment threshold of 6/12 in the better eye^16^ is used to establish need as well as to establish effective correction. Vision impairment is typically reported at the level of a person rather than for each eye separately,^4^, ^17^ so eREC is calculated using visual acuity in the better eye of each individual and reported at the person level.

**Table 1:** Mapping the terms used in the *World Report on Vision* to define effective refractive error coverage by visual acuity measurements and need for refractive error correction

World Report on Vision *(modified* *†* *)*	Visual acuity‐based definitions	Need for refractive error correction
(1) Prevalent cases of vision impairment and blindness due to *uncorrected* refractive error	Individuals with UCVA‡ worse than 6/12 in the better eye who do not have correction and who improve to 6/12 or better with PinVA§	Unmet need (c)
(2) Prevalent cases of *vision impairing* refractive error with spectacles or contact lenses *regardless of visual outcome*	Individuals with UCVA worse than 6/12 in the better eye who have correction and whose CVA**¶**: Is 6/12 or better Improves to 6/12 or better with pinhole over correction	Met need (a) Under‐met need (b)
(3) Prevalent cases of *vision impairing* refractive error with spectacles or contact lenses and a good visual outcome (i.e. do not have vision impairment when wearing spectacles or contact lenses)	Individuals with UCVA worse than 6/12 in the better eye who have spectacles and whose CVA is 6/12 or better	Met need (a)

^†^ Italicised words in column one have been added to the text from the *World Report on Vision* by the authors for clarification.

^‡^ UCVA = uncorrected visual acuity: VA measured with the naked eye/ without correction.

^§^ PinVA = pinhole visual acuity: VA measured with pinhole occluder, either in front of the naked eye or person's own habitual correction.

^¶^ CVA = corrected visual acuity: VA measured with person's own habitual correction.

**Figure 1:**
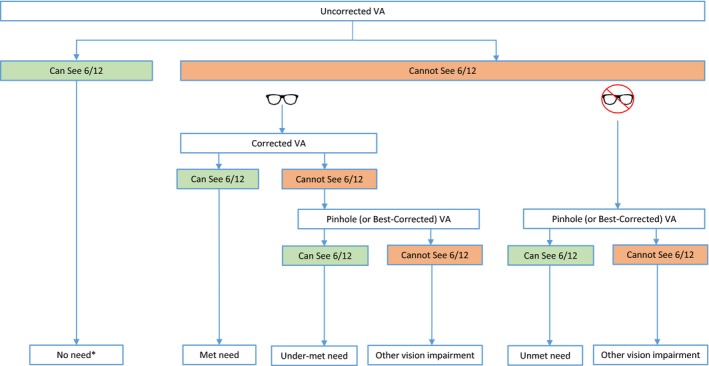
Flow chart demonstrating the visual acuity measurements required to categorise individuals as having no need, met need, under‐met need and unmet need. *No need may include people who have correction but can see 6/12 without it. 6/12 threshold refers to better eye acuity; the ‘spectacle’ symbol represents spectacle or contact lens correction

*Uncorrected refractive error* is considered present when uncorrected visual acuity (VA) worse than 6/12 improves to 6/12 or better with pinhole or refraction (*Table* 1). Individuals with uncorrected refractive error are considered to have *unmet need*. Some individuals will have uncorrected VA of worse than 6/12 in the better eye that improves to 6/12 or better with their own correction (spectacles or contact lenses). These individuals have *met need*. Individuals with correction who do not achieve a corrected VA of 6/12 or better, but improve to 6/12 or better with pinhole (pinhole VA) over their habitual correction or with new refraction (best‐corrected VA), are considered to have *under‐met need*. Anyone with uncorrected VA of 6/12 or better in the better eye is considered to have *no need* for refractive error correction. People wearing refractive error correction, but unable to achieve 6/12 or better in the better eye with the addition of pinhole to their correction will be considered as having *other vision impairment* – a cause other than uncorrected refractive error, e.g., cataract. These individuals are not included in the group with need for refractive error correction. *Need for refractive error correction* is considered as those who have vision impairing refractive error, being the sum of those whose needs are met, under‐met and unmet (*Table* 1 and *Figure* 1). Near visual acuity and need for near vision/presbyopic correction are not included in eREC calculations.

In some contexts, it may be appropriate for the *threshold of need* to be higher or lower than 6/12. For example, cataract surgical coverage (CSC) and effective cataract surgical coverage (eCSC) are typically reported at three levels of cataract‐related vision impairment‐<6/18, 6/60 and 3/60‐depending on the health system context and eligibility criteria for surgery. Here, we define eREC with a 6/12 threshold, but other thresholds for need could be measured and reported depending on the setting and population e.g. 6/18 or 6/9. Regardless of the primary threshold used, to allow for international comparison we propose that all studies that report eREC report results at the 6/12 need threshold.

We have also used 6/12 as the *threshold of a ‘good’ visual outcome* with refractive error correction, the measure of service effectiveness/quality. In some contexts, it may be appropriate for this threshold to be lower (e.g. 6/9 or 6/6), but regardless of the lowest threshold reported, all studies reporting eREC should also report at the 6/12 outcome threshold to allow for international comparison.

Using the VA‐based definitions, eREC can be calculated as follows:

$$\text{eREC}\;\left(\%\right)=\frac{\text{Met}\;\text{Need}\;\left[a\right]}{\left(\text{Met}\;\text{Need}\;\left[a\right]+\text{Undermet}\;\text{Need}\left[b\right]+\text{Unmet}\;\text{Need}\;\left[c\right]\right)}\times 100$$

## eREC: A worked example

Within a survey sample:

50 people have unmet need (*c*)

50 people have distance correction. Of these:

- 20 have distance correction, but have UCVA 6/12 or better (i.e. not vision impaired without correction; excluded from the numerator and denominator)
- 30 people have distance correction and UCVA < 6/12. Of these:
  o 5 have CVA < 6/12 and Pinhole VA ≥ 6/12 (*b*)
  o 25 have CVA ≥ 6/12 (*a*)

$$\text{eREC}\;\left(\%\right)=\frac{\left(a\right)}{\left(a+b+c\right)}=\frac{\left(25\right)}{\left(25+5+50\right)}=\frac{\left(25\right)}{\left(80\right)}\times 100=31\%$$

## Measurement

The purpose of an eye care coverage indicator is to quantify the proportion of a population with an eye health need that has had that need met. As such it must be reported from a representative sample of a defined population of interest – i.e. via a population‐based survey. The calculation of eREC in a population requires two or three separate VA measurements, depending on whether a person presents with correction.

Many surveys currently measure and report *presenting VA (PVA)*, which measures vision with habitual correction, but does not specify whether a person is wearing correction. Surveys wishing to report eREC must routinely measure (1) *uncorrected VA (UCVA)*, (2) *corrected VA (CVA)* for those wearing correction and (3) when either UCVA or CVA <6/12 *pinhole VA (PinVA)* or *best‐corrected VA (BCVA) *when refraction is done. Pinhole VA tends to be more commonly reported as conducting refraction in surveys has extensive resource implications, while pinhole screening has been shown to be effective at identifying refractive error in general populations.^18^, ^19^ These VA measurements will enable estimates of no need, met need, under‐met need and unmet need (*Figure* 1).

## Other considerations

### Identifying the quality gap in refractive error services

In the absence of co‐morbidity, 100% of optical corrections dispensed should give a better eye visual outcome of 6/12 or better. However, within populations there are individuals who wear correction but do not see 6/12 or better, and therefore have under‐met need. There are several causes of under‐met need, including:

- Poor quality refraction
- Poor quality glazing/dispensing
- Damaged spectacle lenses
- A change in prescription since the previous correction was dispensed

The last two causes do not necessarily reflect the quality of the refraction service, but may rather reflect whether services are available, accessible, affordable or acceptable. When a survey identifies a high proportion of participants with under‐met need, the causes could be investigated and findings used to develop appropriate interventions to address identified short‐comings in refractive error services.

By including under‐met in the numerator of the eREC calculation, we arrive at a definition for *refractive error coverage* (REC). REC measures whether vision‐impairing refractive error has been corrected, regardless of whether a ‘good’ outcome is achieved, i.e., it measures the UHC element of access to refractive error correction, but not the element of quality.

$$\text{REC}\;\left(\%\right)=\frac{\text{Met}\;\text{Need}\;\left[a\right]+\text{Undermet}\;\text{Need}\;\left[b\right]}{\left(\text{Met}\;\text{Need}\;\left[a\right]+\text{Undermet}\;\text{Need}\;\left[b\right]+\text{Unmet}\;\text{Need}\;\left[c\right]\right)}\times 100.$$

Returning to the eREC worked example above, REC is higher than eREC:

$$\text{REC}\;\left(\%\right)=\frac{\left(a+b\right)}{\left(a+b+c\right)}=\frac{\left(25+5\right)}{\left(25+5+50\right)}=\frac{\left(30\right)}{\left(80\right)}\times 100=38\%$$

The relative gap between REC and eREC can be calculated to determine the extent of refractive error correction that is under‐met i.e. the Relative ‘Quality’ Gap in refractive error services.

$$\begin{array}{cc} \text{Relative}‘\text{Quality}’\text{Gap}\left(\%\right) & =1-\frac{\left(\text{eREC}\right)}{\left(\text{REC}\right)}\\ & =1-\frac{\left(31.3\right)}{\left(37.5\right)}=17\% \end{array}$$

In survey data from Australia, South Africa and Pakistan, unmet and under‐met need were reported separately, so the quality gap can be calculated (*Table* 2).^8^, ^20^, ^21^

**Table 2:** Comparison of coverage and effective coverage in selected population‐based surveys

Study	Methodology	Age Group (years)	WHO Region	Country	eREC (reported by study)	REC (calculated from text)	Quality gap in refractive error services†
Naidoo (2016)	Sub‐national; RARE	15‐35	Africa	South Africa	51.4%	54.3%	5.3%
Shah (2008)	National eye health survey	30+	South‐East Asia	Pakistan	15.1%	22.7%	33.5%
Foreman (2017)	National eye health survey	40+	Western Pacific	Australia	93.5% (Non‐Indigenous) 82.2% (Indigenous)	98.7% 94.0%	5.3% 12.0%

eREC, effective refractive error coverage, WHO, World Health Organization.

^†^ The relative gap between eREC and REC is calculated as (1 – (eREC/REC)).

## Non‐compliance with refractive error correction

Non‐compliance with prescribed refractive error correction is a concern, particularly among children.^22^ As eREC is derived from population‐based surveys, anyone not habitually wearing their correction at the time of data collection will be categorised as having unmet need, i.e., non‐compliance will not be detected. We recognise that there is a need to explore non‐compliance as a barrier to met need.

## eREC targets

The WHO has not yet set a specific target for the 2023 Milestone pertaining to the coverage of essential health services.^23^ It has previously recommended that each country set its own UHC targets based on local priorities and realities and this was reaffirmed in the *World Report on Vision*. The need for local eREC target‐setting becomes evident given the large range in refractive error or spectacle coverage previously reported – from over 90% in non‐Indigenous Australians,^8^ to around 50% in urban Colombia,^10^ to <5% in Nigeria.^7^

## Reporting

We propose that REC and eREC are both reported from population‐based surveys along with the proportions and sample numbers with no need and met, unmet and under‐met need for refractive error correction. We propose that studies report how they defined refractive error correction, i.e., spectacles ± contact lenses. Sample proportions can be extrapolated to the population using population data, e.g., from a census. Where surveys report age and sex adjusted estimates (on account of non‐representativeness of sample) eREC should also be adjusted.

## Presbyopic correction coverage

The *World Report on Vision* highlighted the economic impact of the decreased productivity associated with as many as 800 million people having uncorrected or under‐corrected presbyopia, alongside the one billion with corrected presbyopia.^4^ Presbyopic spectacle coverage has previously been reported alongside, but separate to, refractive error or spectacle coverage.^10^, ^11^, ^13^, ^15^, ^24^, ^25^ We believe the need for refractive error correction and presbyopic correction should continue to be reported separately due to differences in (1) the need for refractive error correction in different populations, (2) the measurements required for the two conditions and (3) the implications for services. To improve monitoring of this vast eye health need, standardised definitions, methods and reporting of presbyopic need and coverage in population‐based surveys is required.

## Conclusion

The *World Report on Vision* highlighted the need for consensus on the definition and measurement of eye health indicators, and emphasized the importance of effective coverage indicators for refractive error and cataract.^4^ Here we have provided a detailed outline of how effective refractive error coverage (eREC) can be measured and calculated.

eREC is an indicator of the availability, accessibility, affordability and acceptability of refractive error services provided in a defined area. Baseline and follow‐up population‐based measurements of effective coverage can inform eye health planners about progress towards improving the access to, and quality of, their services.

Standardised definitions, methods and reporting of refractive error correction need and eREC – disaggregated by sex, place of residence, socioeconomic position and disability^26^ wherever possible – will improve our understanding of eye health need in populations, enable evidence‐based planning for eye health services and, ultimately, assist the realisation of universal health coverage.
